# NQR sensitive embedded signatures for authenticating additively manufactured objects

**DOI:** 10.1038/s41598-021-91531-6

**Published:** 2021-06-09

**Authors:** Naren Vikram Raj Masna, Junjun Huan, Soumyajit Mandal, Swarup Bhunia

**Affiliations:** Department of Electrical and Computer Engineering, University of Florida, Gainesville, FL USA

**Keywords:** Electrical and electronic engineering, Electronic devices, Design, synthesis and processing, Imaging techniques, Solid-state NMR

## Abstract

Automatic recognition of unique characteristics of an object can provide a powerful solution to verify its authenticity and safety. It can mitigate the growth of one of the largest underground industries—that of counterfeit goods–flowing through the global supply chain. In this article, we propose the novel concept of *material biometrics*, in which the intrinsic chemical properties of structural materials are used to generate unique identifiers for authenticating individual products. For this purpose, the objects to be protected are modified via programmable additive manufacturing of built-in chemical “tags” that generate signatures depending on their chemical composition, quantity, and location. We report a material biometrics-enabled manufacturing flow in which plastic objects are protected using spatially-distributed tags that are optically invisible and difficult to clone. The resulting multi-bit signatures have high entropy and can be non-invasively detected for product authentication using $^{35}$Cl nuclear quadrupole resonance (NQR) spectroscopy.

## Introduction

Counterfeiting of popular branded products is an old phenomenon. Although there is growing public awareness of counterfeit products, there is limited availability of authentication devices in the market. Counterfeit items can reach the customer in numerous ways including online sales, street markets, and dishonest marketers. There is also the possibility of fake items infiltrating a legitimate supply chain, thereby compromising its integrity^1^. The gravity of the situation can be gauged from the variety of well-funded anti-counterfeit programs that are being launched by companies in multiple business sectors. According to preliminary data collected by the International Trademark Association, the total cost of fakes exceeded $500 billion in 2019^2^. Counterfeit goods not only cause damage to our economy, they also pose significant health and safety risks. Growing popularity of e-commerce and consumer habits of online purchases are aggravating the problem. To mitigate the flow of counterfeit and pirated goods, Customs and Border Protection (CBP) and U.S. Immigration and Customs Enforcement’s Homeland Security Investigations (HSI) recently created an aggressive enforcement program. In the year 2014 alone, they reported 23,000 seizures of fake products worth an estimated $1.2 billion. These products could have threatened the health of consumers and resulted in revenue loss for many legitimate companies. Some of the most commonly counterfeited products include consumer electronics, optical media, apparel/accessories, handbags/wallets, footwear, watches/jewelry, pharmaceuticals/personal care, toys and computers/accessories. Most of these products are at least partially constructed out of various grades of plastic. Ironically, labels and tags made from plastic—particularly for designer goods—are themselves in the list of ten most counterfeited products^3^.

There have been many instances of counterfeiting during the complicated times of the COVID-19 pandemic. Counterfeiters have even taken advantage of high demand to manufacture copies of patented medical devices like ventilators, N95 masks, and other PPE equipment that are required for intensive care and frontline workers^4^. Additive manufacturing (i.e., 3D-printing) technology has been a boon in making medical devices^5^. However, increased accessibility to 3D-printing technology has also led to increased counterfeiting, thus creating uncertainty among frontline workers and reducing trust in legitimate supply chains. Even concerned people with no malicious intent who try to use 3D-printing to help address the demand for medical devices are likely doing more harm than good^6^.

Earlier work on minimizing the effect of counterfeit products has concentrated on supply chain management, not consumer protection. The dominant approach relies on add-on tags based on the universal product code (UPC), quick response (QR) patterns, radio-frequency identification (RFID), or nanoelectromechanical (NEMS) resonators^7^. Attempts have also been made to use chemical authentication techniques based on add-on nucleic acid tags. In this process, solid substances are tagged by spreading a water-insoluble medium containing known nucleic acids on the surface. Similarly, liquids are tagged by mixing them with the same water-insoluble medium^8^. Built-in watermarks constitute an alternative authentication approach. For example, pseudo-watermarks on printable, flexible, and synthetic supports have been proposed for authenticating plastics. Each support bears at least one authentication or security mark, such as a synthetic substrate material on one face that can be identified via altered opacity^9^. Finally, the molecular properties of materials within the product can be used as intrinsic watermarks (i.e., chemical fingerprints). Examples include authentication of pharmaceutical products through different types of packaging using nuclear quadrupole resonance (NQR) spectroscopy^10, 11^ and spatially-offset Raman spectroscopy (SORS)^12^.

The aforementioned methods for authenticating products have various disadvantages. Add-on tags are easily removed, cloned, tampered with, and/or reapplied by counterfeiters. Built-in watermarks are harder to remove, but have not been demonstrated to store enough unique information to prevent cloning. Direct chemical fingerprinting is highly product-specific and cannot be applied to materials without easily-read signatures. Material biometrics is a potential solution to these long-standing problems, since it creates unique chemical signatures that are invisible to attackers, embedded within the structure (and thus impossible to remove without major damage), and can be read non-invasively. Moreover, signature generation can be integrated within an automated additive manufacturing flow, thus enabling widespread deployment of the proposed approach. The signatures are generated by embedding spatially-distributed tags (containing a chemically-responsive material) within the physical structure of the product. Each signature utilizes multi-modal information (consisting of the locations, chemical compositions, quantities, and local environments of the tags) to ensure uniqueness and unclonability. Signatures can be non-invasively read out using a variety of detection methods, including NQR, Raman spectroscopy, and near-infrared (NIR) spectroscopy. In this paper, the authentication process relies on NQR spectroscopy of molecules containing quadrupolar nuclei. NQR-based chemical signatures are quantitative and highly-specific, and can be detected using non-invasive, non-destructive, and low-cost instrumentation^10, 11^. Unlike optical methods such as NIR and Raman spectroscopy, NQR is a radio frequency (RF) technique that is insensitive to packaging, opacity of the material, surface roughness, and other physical characteristics.

**Figure 1:**
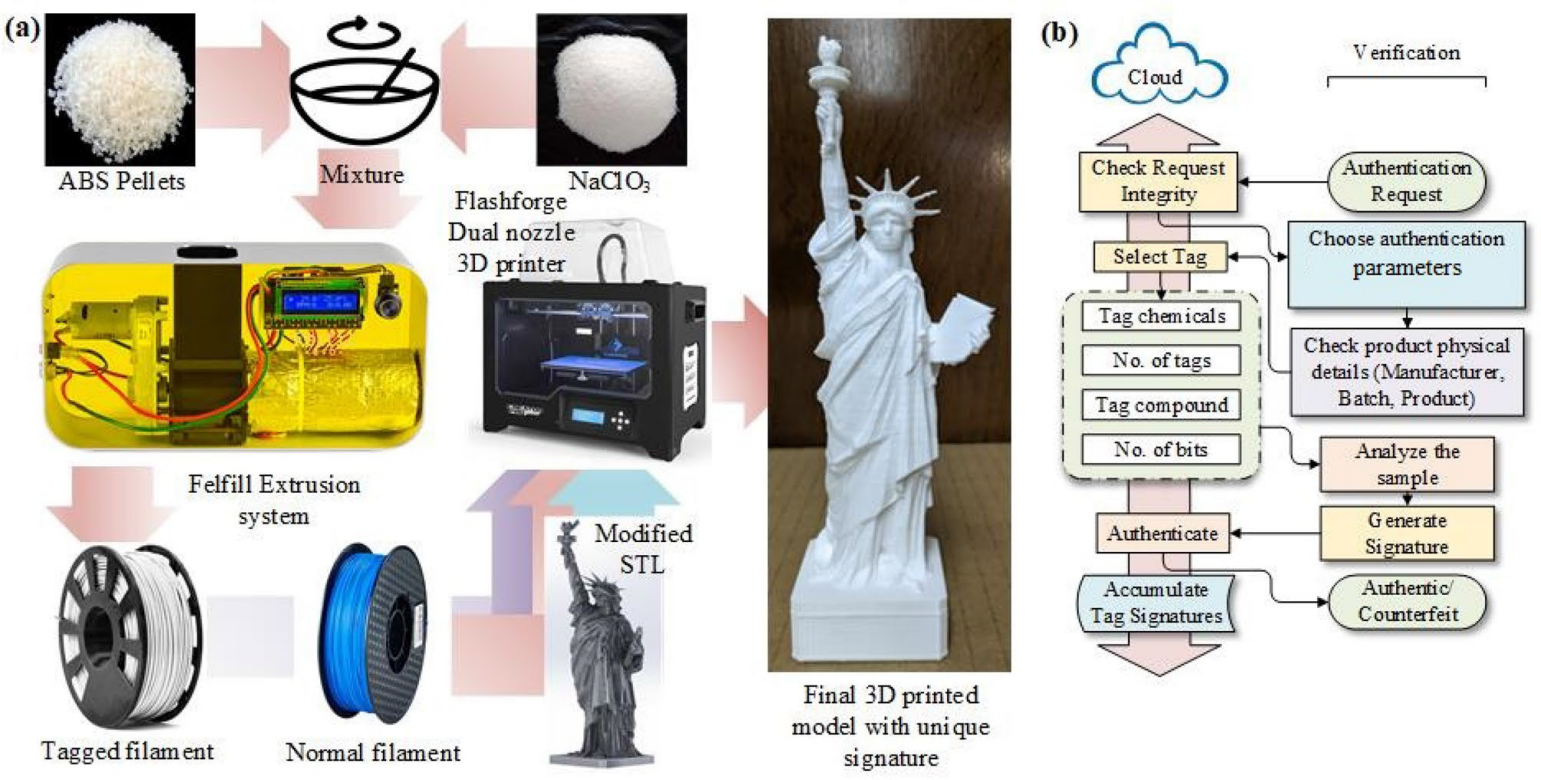
(**a**) Overview of the signature generation process for material biometrics. Custom tagged filament is created by mixing acrylonitrile butadiene styrene (ABS) with an NQR-sensitive material. Tagged and normal (un-tagged) filaments are dispensed by a dual-filament 3D printer to generate arbitrary 3D spatial tagging patterns within the printed object. (**b**) Flowchart of the general process for product authentication using a cloud database and signature information read from the embedded tags [Microsoft Visio].

We demonstrate the material biometrics concept by integrating it within an additive manufacturing process flow for creating arbitrary plastic objects. As can be seen in Fig. 1a, our method relies on creating custom tagged filaments by mixing raw plastic pellets with a small amount of NQR-sensitive chemical (NaClO$_{3}$ in our case). A filament extrusion system is used to form the mixture into cylindrical filaments that can be fed into a 3D printer. A dual-nozzle printer is loaded with both the modified (i.e., tagged) filament and a normal (i.e., un-tagged) filament. The printer is then loaded with a modified STL (STereoLithography) design that incorporates the desired 3D spatial pattern for the tag. STL is a common file format used for describing the surfaces of objects using triangular facets. The final printed object now contains a unique spatially-varying NQR signature that can be verified using any authentication model of the manufacturer’s choice. The general authentication model shown in Fig. 1b can be customized for product validation at any point in the supply chain as well as by the consumer by utilizing a portable NQR spectrometer and a smartphone that runs an authentication application. Manufacturers need to store the signatures of the tags being used for product validation on a cloud server (ideally, encrypted to maintain integrity of the authentication data against spying and man-in-the-middle attacks). The validation step can either be utilized to authenticate the product or to trace it back to its manufacturer (when labels are damaged or missing). As the proposed multi-modal signatures are extremely difficult to replicate, the products under test can be utilized to check for various integrity issues, e.g., adulteration, mislabeling, and contamination. A validation failure at any stage can be brought to the notice of an appropriate authority (manufacturer, law enforcement agency, etc.) so that appropriate remedial actions can be taken.

## Results

As most common consumer products contain plastic parts, we implemented the proposed material biometrics approach by tagging plastic filaments with an NQR-active compound, namely sodium chlorate ($\hbox {NaClO}_3$). Note that the tagged product need not contain only plastic parts; the inclusion of metal parts does not significantly affect the amplitude, other features, or signal-to-noise ratio (SNR) of the measured NQR signatures^13, 14^.

**Figure 2:**
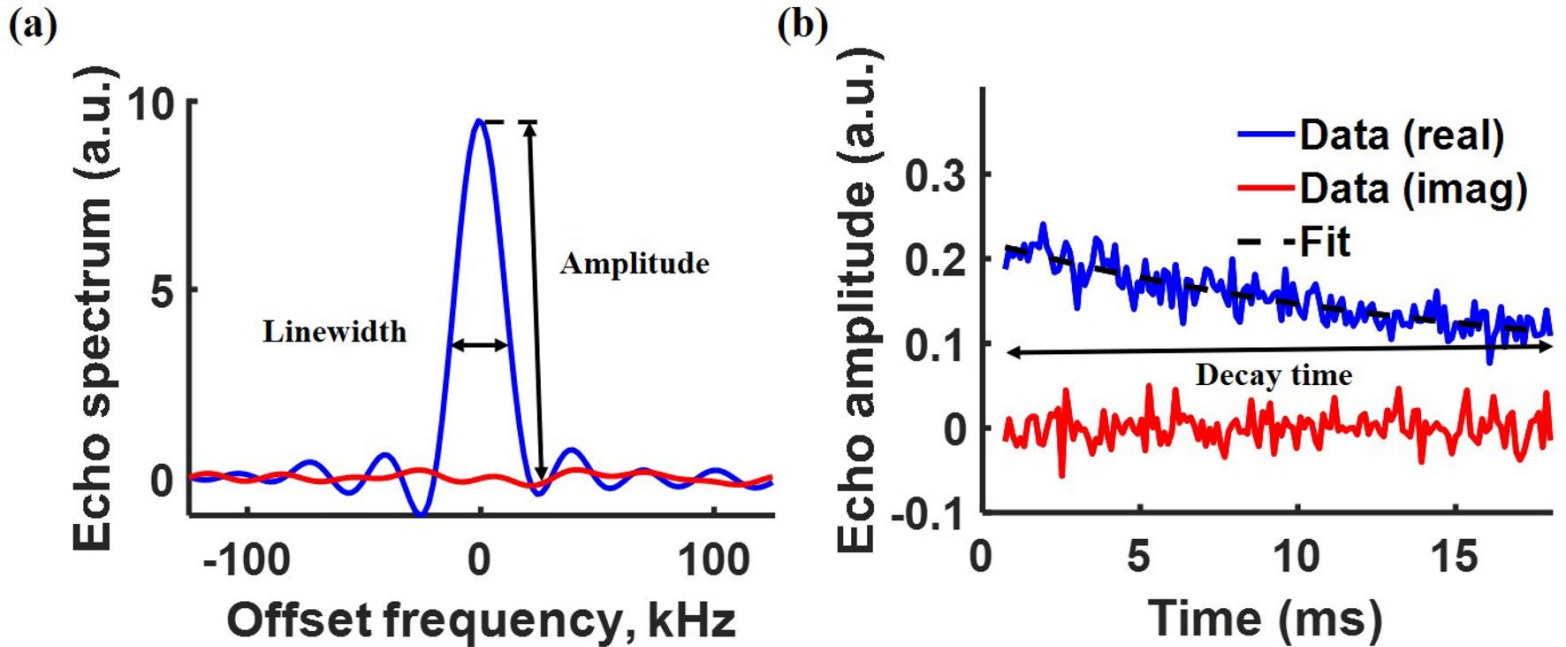
Spin echoes from pulsed NQR measurements using the spin-locked spin echo (SLSE) sequence^15^ are collected, filtered, and fitted to exponential decay curves. Assuming a mono-exponential decay, the resulting function contains three useful features. (**a**) Typical echo spectrum (shown as a function of frequency offset from the nominal resonance frequency). The first feature is the peak signal amplitude *A*, which is proportional to the number of quadrupolar nuclei within the tag. The second feature is the frequency-domain linewidth $\Delta f$. (**b**) Typical echo decay curve. The third feature is the decay time constant $T_{2,eff}$, which defines the time taken by spin coherences to decay after being excited by resonant RF pulses.

### Signature generation

The most important goal of the authentication model is to create a unique signature. We use time- and frequency-domain NQR features of tagging compound(s) to generate the signature of a single tag. A set of such tags is spatially distributed within the object during the additive manufacturing process to greatly increase the length, entropy, and unclonability of the signature, as described later. The amount of tagging compound required to reliably read a single tag depends on its NQR resonance frequencies. Each chemically distinct quadrupolar nucleus within the compound generates multiple (*N*) resonances, depending on its spin *I*; $N=3$ and 1 for $I=1$ and 3/2, respectively. Assuming standard inductive detection of the spin coherences, detection sensitivity and SNR are proportional to $\omega _{0}^{2}$ where $\omega _0$ is the resonant frequency. Thus, higher NQR frequencies allow the use of smaller amounts of tagging compound. For example, detecting a common quadrupolar nucleus such as $^{14}$N, which has $I=1$ and resonance frequencies in the 2–5 MHz range, is significantly more difficult than detecting $^{35}$Cl, which has $I=3/2$ and resonance frequencies in the 25–35 MHz range. Moreover, while compounds containing other quadrupolar nuclei, such as $^{27}$Al, $^{79}$Br, $^{81}$Br, and $^{127}$I, can have very high resonant frequencies, they are generally not suitable for tagging because they (1) have very short signal decays and broad linewidths, which decreases sensitivity; and/or (2) are poisonous, explosive, or otherwise hard to handle. Thus, in this paper we focus on $^{35}$Cl NQR for authentication.

Properties of a good signature read-out method include non-invasiveness, reliability, and rapid data acquisition. We use the spin-locked spin echo (SLSE) multi-pulse sequence^15^ and an inductive detector (i.e., coil) to read out spin echo signals from the chosen NQR-active tagging compound. This pulse sequence consists of an initial RF excitation pulse and a long train of refocusing pulses separated by an echo period. Both the excitation pulse and the refocusing pulses have the same length, with a relative phase shift of $\pi /2$ between them. NQR signals (spin echoes) appear in-between the refocusing pulses^10, 11^. Nonlinear fitting is used to extract three parameters from these echoes: amplitude (*A*), frequency-domain linewidth ($\Delta f$), and relaxation (i.e., decay) time constant ($T_{2,eff}$). We chose sodium chlorate ($\hbox {NaClO}_3$) as our tagging compound since it has high sensitivity, is stable at typical additive manufacturing temperatures ($\sim$ 250 $^{\circ }$C), is widely available, and exhibits magnetic field-dependent relaxation rates (i.e., $T_{2,eff}$ values) that can be used to spatially localize the tags^16^. Typically-observed NQR features of $\hbox {NaClO}_3$ for on-resonance RF pulses at $\omega _{0}=2\pi \times 29.92$ MHz are shown in Fig. 2. The extracted features serve as a unique NQR signature for the tagged plastic part, even though it is not NQR-sensitive by itself. The NQR signature, particularly the linewidth $\Delta f$, also varies with manufacturing process^17, 18^. Thus, any modifications in the physical properties of the sample (e.g., due to manufacturer- or batch-specific processing conditions) tends to further increase the uniqueness of the signature^19^.

**Figure 3:**
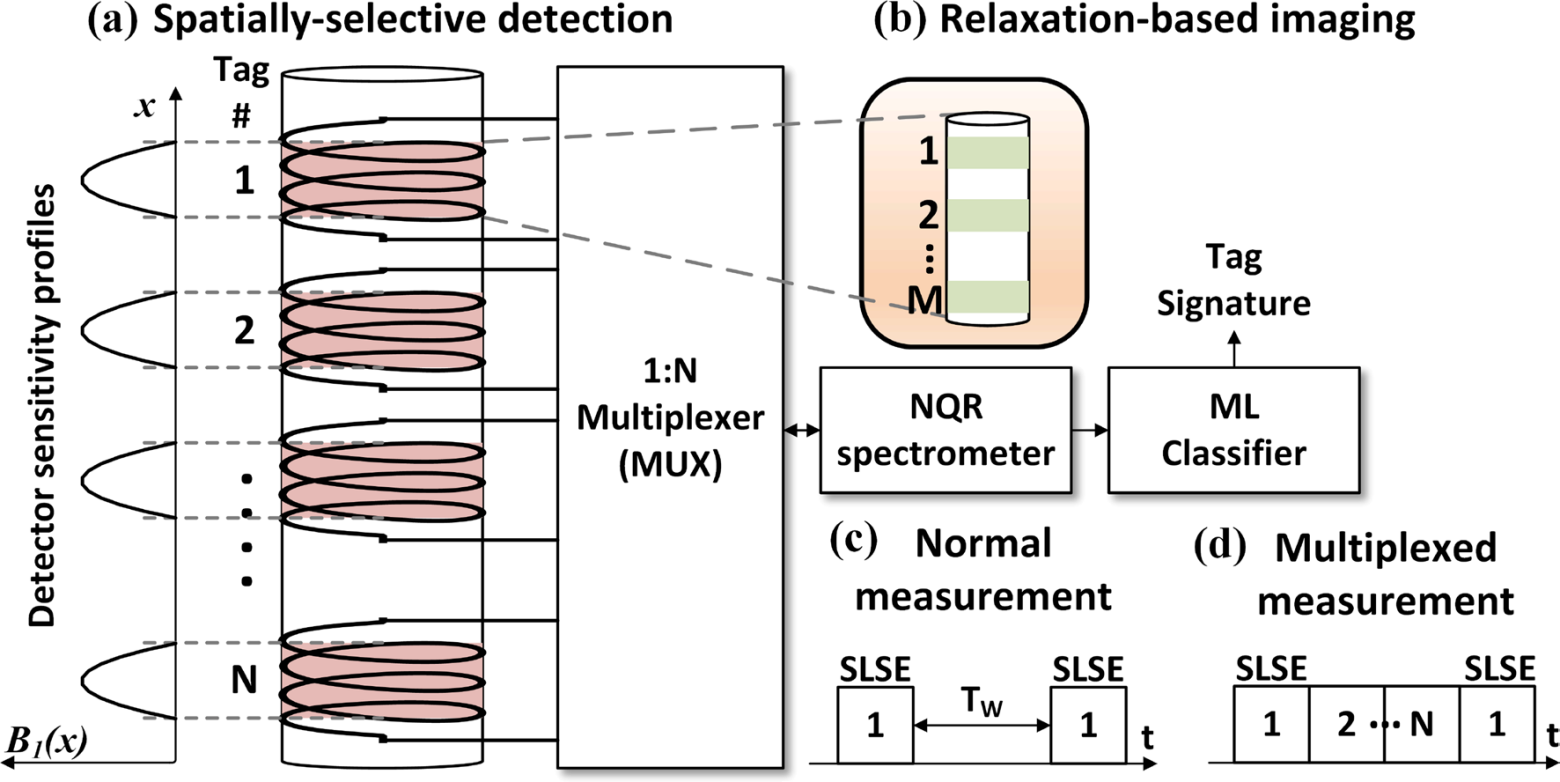
(**a**) Spatially-selective detection using an array of hollow solenoid coils. The system measures signatures from *N* groups of tags near-simultaneously by using a 1 : *N* analog multiplexer. (**b**) Signatures from *M* tags within each group can be measured per scan using relaxation-based imaging. Thus, a total of $N\times M$ tags can be measured by combining the two techniques. (**c**) A normal SLSE pulse sequence for signature read-out which requires a wait-time of $T_w$. (**d**) A multiplexed SLSE pulse sequence that allows signature from other tags to be acquired during $T_w$ [Microsoft Visio].

The read-out time for NQR signatures depends on the experimental setup. We explored two innovative concepts for minimizing read-out times, given a certain signature geometry (i.e., number, size, and separation of embedded tags): *spatially-selective detection* (Fig. 3a) and *relaxation-based imaging* (Fig. 3b).

**Figure 4:**
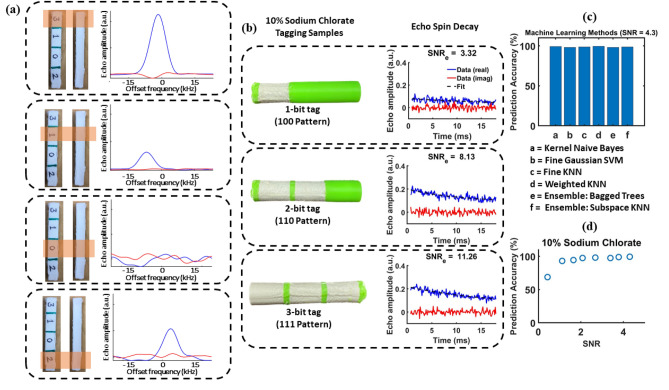
(**a**) Signatures from specific parts of the sample are read out using a spatially-selective “hollow coil” detector; each signature has a different value of the peak amplitude *A*. The number on the label represents the relative amount of $\hbox {NaClO}_3$ used for tagging. (**b**) Cylindrical samples containing three tagging locations, each using 10% $\hbox {NaClO}_3$ filament. The figure shows three of the possible binary patterns and their field-dependent relaxation decays. (**c**) Signature prediction accuracy of different machine learning (ML)-based classification methods (listed on the figure). (**d**) Measured improvement in prediction accuracy with increase in SNR for 3-bit patterns (10% $\hbox {NaClO}_3$) [Microsoft Visio].

### Spatially-selective detection

For simplicity, we assume a 1D distribution of tags within the sample. A set of *N* spatially-selective detectors is used to simultaneously measure signatures from these tags, thus reducing the total measurement time by a factor of *N*. The detectors consist of a 1D array of hollow solenoid coils, each of which has a localized sensitivity profile as shown in Fig. 4a. While an *N*-channel spectrometer can be used to interface with these detectors, the resulting hardware costs become prohibitive as *N* increases. Instead, here we use time-domain multiplexing. A normal NQR signature measurement requires wait times $T_{W}$ for longitudinal relaxation between successive SLSE sequences, as shown in Fig. 3c. In our multiplexed version, an analog multiplexer is used to measure signatures from other detectors during these wait times (Fig. 3d).

Our experimental prototype uses detectors that are 2 cm long and have a diameter of 1 cm. For initial tests, we used a plastic sample (8 cm in length) divided into four parts (each 2 cm long). Each part contains an embedded tag whose $\hbox {NaClO}_3$ concentration can be varied during the additive manufacturing process and read out by a single detector. Figure 4a shows measured data when the concentration is set to one of 4 values, thus resulting in 2-bit amplitude information per tag. The figure confirms that this information can be read out from the measured echo spectra. Together, the 4 tags thus act as a complex (and optically invisible) chemical identifier with a 8-bit amplitude signature. Other NQR signal features, such as linewidth and decay rate, can be used to augment the signature length.

Information required for authenticating such signatures, such as tag length, position, and concentration, are securely stored in the cloud and can be accessed only by authorized users as shown in Fig. 1a. The process is automated by linking signature records in the cloud database to publicly-available product identifiers (e.g., UPC, QR code, or RFID) that can be read using off-the-shelf devices (optical bar code scanners, cameras, or RFID readers).

### Relaxation-based imaging

The amount of multiplexing possible using spatially-selective detection is limited by the fact that the sensitivity functions become poorly localized as coil length decreases (the minimum useful length/diameter ratio for solenoids is $\sim$ 2). Thus, further increases in tag number (and hence the signature length) requires multiple tags to be read out from a single detector. We use relaxation-based NQR imaging^16^ for this purpose. Unlike earlier NQR imaging methods^20^, relaxation-based imaging does not require multiple scans for indirectly encoding of spatial information; thus, it is a rapid or “single-shot” technique.

Relaxation-based imaging relies on the fact that the SLSE relaxation rate $T_{2,eff}$ of several chlorine compounds, including $\hbox {NaClO}_3$, depends on the magnitude of the local static magnetic field (denoted by $\left| B_0\right|$). Thus, applying a 1D or 2D field gradient across the sample creates a spatially-varying relaxation profile. The latter can be inverted to yield the sample’s spin density distribution, thus generating a 1D or 2D image of the tags. Here we focus on using 1D imaging to simultaneously read out *M* tags from each detector, as shown in Fig. 4a. Instead of directly generating images (which requires the use of an inverse Laplace transform, which is numerically ill-conditioned for noisy measurements^21^), we use a machine learning (ML) approach. In particular, a trained ML classifier is used to determine which of the $2^{KM}$ possible amplitude signatures (assuming *K* amplitude bits per tag) best matches the measured time-domain data.

The tagged samples used for the experiments were 3D printed using a multi-nozzle printer, as shown in Fig. 1a. The first nozzle dispenses regular acrylonitrile butadiene styrene (ABS) filament (for printing untagged parts of the sample), while the others dispense custom ABS filaments containing small amounts of crystalline $\hbox {NaClO}_3$ (for printing tags). Achieving *K*-bit amplitude control over the tags requires $2^{K}$ custom filaments, each with a different $\hbox {NaClO}_3$ content, and thus a total of $2^{K}+1$ nozzles. For simplicity, here we use single-bit amplitudes ($K=1$), for which a dual-nozzle printer is sufficient.Thus, the filaments are used to make bit patterns, where tagged filament represents ‘1’, and untagged filament (at tag locations) represents ‘0’. A few 3D-printed cylindrical samples containing various 3-bit patterns are shown in Fig. 4b. The tagged (10% $\hbox {NaClO}_3$) and untagged filaments have different colors to aid visualization; otherwise, they would be visually indistinguishable, as in Fig. 4a.

Our experimental setup is designed to accommodate $M=3$ tags per detector, resulting in $2^{3}=8$ possible bit patterns. Each tag was designed to be 1.2 cm long (to ensure adequate SNR) with inter-tag gaps of 2 mm (to ensure adequate classification accuracy). Field gradients for relaxation imaging were generated using Helmholtz coils (as described later). A few of the resulting time-domain decay curves are shown in Fig. 4b. A set of curves from known samples of this type were used to train an 8-class ML classifier.

Classical classification techniques (e.g., for image processing) are based on template matching^22, 23^. In this approach, a $T_{2,eff}$ distribution is used as a template for each class. Measured signatures are correlated with each template (either in the time- or frequency-domain), and the best-matching template (i.e., with the highest cross-correlation coefficient) is selected as the class. However, classification accuracy is subject to optimal selection of the template. Instead, here we used ML models to rapidly and accurately classify the measured signatures. Classification accuracy increases with SNR, which can be improved by signal averaging at the cost of measurement time. Specifically, SNR $\propto \sqrt{N_{av}}$ where $N_{av}$ is the number of scans. We trained classification models at several SNR levels by collecting time-domain relaxation data (150 echoes) for various values of $N_{av}$ from samples with known tagging patterns. The right-hand column of Fig. 4b shows measured data for the three example patterns shown in the left-hand column. Principal component analysis (PCA) was used as a feature extraction method, i.e., to reduce data dimensions prior to classification. The prediction accuracy (*PA*) of a variety of ML models was then estimated via cross validation. Figure 4c shows *PA* for six different models using 3-bit tagged samples (10% $\hbox {NaClO}_3$) with a SNR of 4.3 per echo. The results demonstrate that all six ML models have very high *PA* (over 98%) in this case. The “fine KNN” method (*k*-nearest neighbors algorithm with $k=1$) was selected as the best-performing classification model since it has the highest *PA* of 99.4%. Figure 4d shows *PA* for the fine KNN method as a function of SNR. The figure shows that *PA* $\sim$ 100% for SNR $>2$.

**Figure 5:**
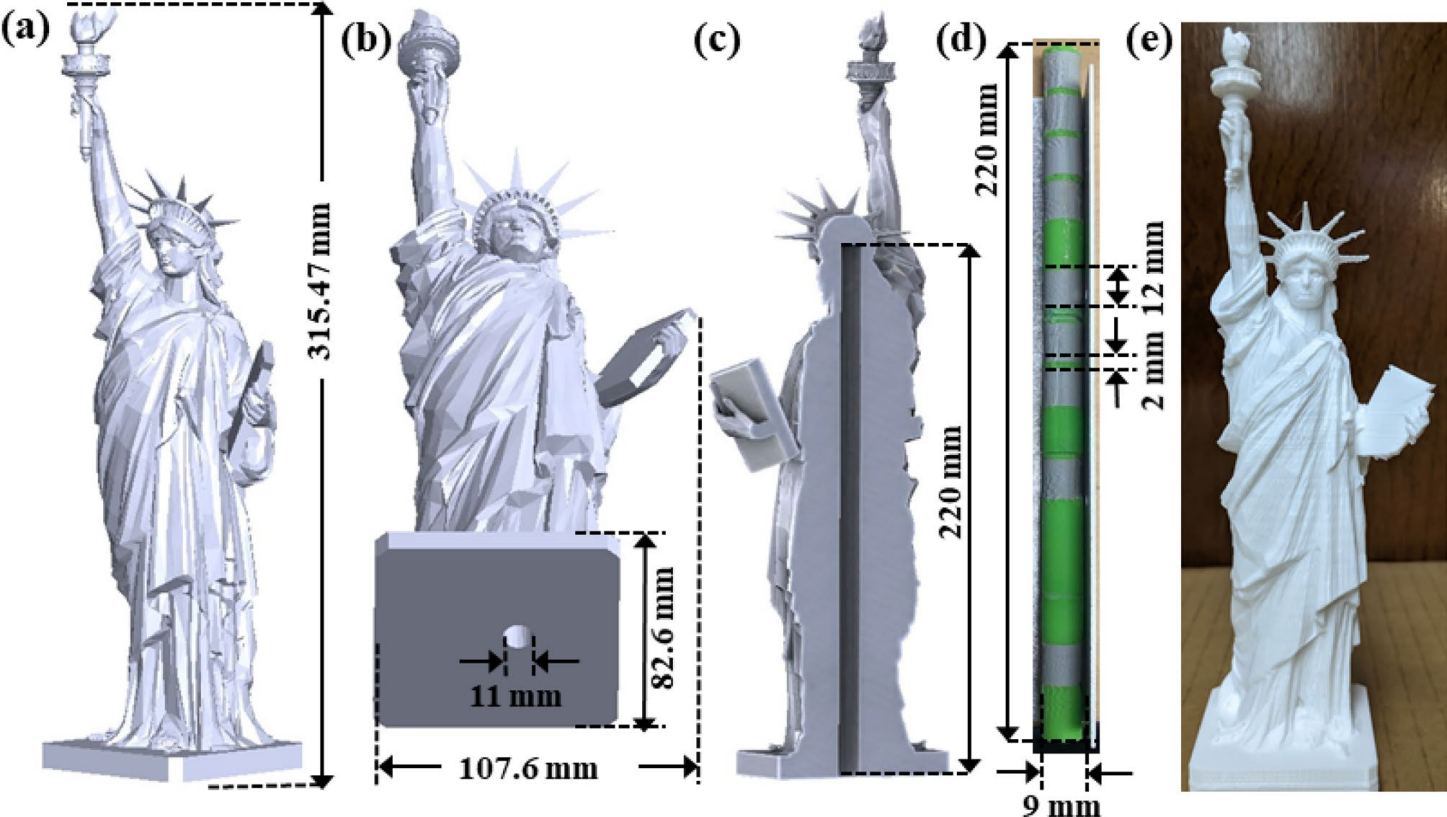
(**a**) The original STL design of “the Statue of Liberty”. (**b**) The modified design used to embed an NQR-sensitive signature within the object. (**c**) A cross-sectional view of the modified design, showing the space to be occupied by the tags. (**d**) A typical signature pattern printed using two different colors (white representing tagged filament, and green representing regular filament). The signature contains 15 tags, with each tag encoding $K=1$ amplitude bit. The modified object and signature can either be printed together, or the latter can be inserted as an add-on after printing the object. (**e**) The resultant 3D-printed object containing the embedded material biometrics signature [Microsoft Visio].

### Sensitivity and limit of detection

The sensitivity of the proposed NQR-based tagging and signature read-out method can be theoretically estimated as follows. We begin by estimating the magnetization density within the tag immediately after an RF excitation pulse with a nutation angle of $\theta$. Assuming that (1) the sample was originally in thermal equilibrium, and (2) the RF pulse has enough bandwidth to excite the entire NQR linewidth, the result is^24^

1 $$\begin{aligned} {M_{0}(\theta )=s_{exc}(\theta )\rho _s\frac{2}{3}\frac{\gamma \hbar ^2\omega _0}{(2I+1)k_{B}T_s}\left( \frac{I(I+1)-mm^{\prime }}{I^2}\right) , }\end{aligned}$$

where $s_{exc}(\theta )$ is known as the nutation function, $\rho _s$ is the spin density (i.e., the number of $^{35}$Cl nuclei per unit volume), $\gamma$ is the gyromagnetic ratio of the nucleus, $\hbar$ is the reduced Planck’s constant, $k_{B}$ is Boltzmann’s constant, and *m* and $m^{\prime }$ are the spin quantum numbers of the two nuclear states involved in the NQR transition. Due to quantum selection rules, these values must satisfy $|m-m^{\prime }|=1$. For spin-3/2 nuclei such as $^{35}$Cl, the positive- and negative-valued states (i.e., $m=\pm 1/2$ and $m^{\prime }=\pm 3/2$) are degenerate in the absence of an external $B_0$ field, so both of the allowed transitions ($-1/2 \leftrightarrow -3/2$ and $+1/2 \leftrightarrow +3/2$) occur at the same resonant frequency. The factor of 2 in Eq. (1) takes both these transitions into account.

For polycrystalline tagging materials (such as the NaClO$_3$ powder used in this study), the nutation function $s_{exc}(\theta )$ must be calculated via a so-called powder average over all possible relative orientations of the crystallographic principal axes to the RF magnetic field generated by the pulse, $\mathbf {B}_{1}$. The result can be shown to be^25^

2 $$\begin{aligned} {s_{exc}(\theta )\approx \frac{2}{\sqrt{3}}J_{1}(\theta ), }\end{aligned}$$

where $J_1(\theta )$ denotes the Bessel function of the first kind. The peak value of this function is $\approx 0.672$, which occurs at the optimum nutation angle of $\theta _{opt}\approx 1.84$ rad (i.e., $105^{\circ }$). Finally, the spin density $\rho _s$ can be estimated as

3 $$\begin{aligned} {\rho _s = \Phi _v\left( \frac{c_{35} N_s N_A \rho }{MW}\right) , }\end{aligned}$$

where $\Phi _v$ is the volume fraction of the tag (NaClO$_3$ in this case), $c_{35}$ is the isotopic abundance of the active nucleus (approximately 75.5% for $^{35}$Cl), $N_{s}=1$ is the number of equivalent nuclear sites in the tag molecule (all of which would contribute to the observed transition), $N_A$ is Avogadro’s constant, $\rho \approx 2.5$ g/cm$^3$ is the bulk density of the tag, and $MW\approx 106.44$ is its molecular weight. The volume fraction can be estimated as $\Phi _v = \Phi _w\times (\rho _{av}/\rho )$, where $\Phi _w$ is the weight fraction of the tag (typically 10% in our case) and $\rho _{av}$ is the average density of the tagged object. The latter is given by $\rho _{av}=\Phi _w\rho +(1-\Phi _w)\rho _{m}$, where $\rho _{m}\approx 1.04$ g/cm$^3$ is the bulk density of the original object (ABS in our case).

The induced magnetization, $M_0$, represents the initial amplitude of a quantum coherence that oscillates sinusoidally at the resonant frequency, $\omega _0$. By Faraday induction, the resulting time-varying magnetic flux generates a RF voltage in the detector coil. Using the principle of reciprocity for electromagnetic fields^26^, the amplitude of this voltage is given by

4 $$\begin{aligned} {V_{coil}=\omega _0\int _{V_s}\left( \frac{B_1(\mathbf {r})}{I_1}\right) M_0(\theta (\mathbf {r}))dV_{s}, }\end{aligned}$$

where the integral is carried out over $V_{s}$, the sample volume; $\theta (\mathbf {r})=\sqrt{3}B_{1}(\mathbf {r})t_p$ is the position-dependent nutation angle (with $t_p$ being the RF pulse length); and $B_{1}(\mathbf {r})/I_{1}$ is the position-dependent *coil sensitivity function*, i.e., the amplitude of the RF field generated by a unit current flowing in the coil. Typically, we optimize the spatial homogeneity of $\theta$ (and thus $M_{0}$) by adjusting $t_p$ such that $\theta (\mathbf {r})=\theta _{opt}$ when $B_{1}=\overline{B_{1}}$, its average value within the sample.

The coil sensitivity function, which is a measure of coupling strength between the coil and the sample, can be estimated for a given coil geometry by using the Biot–Savart law. We used solenoid coils of length $l_c$, diameter $D_c$, and $N_c$ turns in our experiments. The $B_1$ field within these coils is nearly uniform in planes transverse to the coil axis (denoted by *x*) when $l_c > D_c$ (as in our designs), and the coil sensitivity function can then be analytically derived (using a current sheet approximation) as

5 $$\begin{aligned} {\frac{B_1(x)}{I_1}=\frac{\mu _0 N_c}{2l_c}\left( \frac{2x+l_c}{\sqrt{\left( 2x+l_c\right) ^2+D_c^2}}-\frac{2x-l_c}{\sqrt{\left( 2x-l_c\right) ^2+D_c^2}}\right) , }\end{aligned}$$

where $\mu _0 = 4\pi \times 10^{-7}$ H/m is the vacuum permeability. Note that this formula reduces to the well-known result $\frac{\mu _0 N_c}{l_c}$ when $l_{c}\gg D_{c}$, i.e., for an infinitely-long solenoid. Equation (5) can be substituted in Eq. (4), resulting in a 1D integral (with $dV_{s}$ replaced by $A_{s}dx$ where $A_{s}$ is the cross-sectional area of the sample) that can be numerically evaluated to estimate $V_{coil}$. Ignoring relaxation, the SNR per echo (in power units) is then given by

6 $$\begin{aligned} {SNR_{e} = \frac{V_{coil}^{2}}{2\sigma ^{2}_{n}}, }\end{aligned}$$

where $\sigma _{n}$ is the root-mean-squared (rms) noise in the coil and the factor of 2 arises from converting amplitude to rms. In the absence of external sources (such as RFI), $\sigma _{n}$ is dominated by the thermal noise of the coil and given by

7 $$\begin{aligned} {\sigma ^{2}_{n}=4k_{B}T_{c}R_{c}F\Delta f, }\end{aligned}$$

where $T_c$ is the coil temperature (here assumed to be equal to $T_s$, the sample temperature), $R_c$ is the series coil resistance, $F>1$ is the noise factor of the receiver, and $\Delta f$ is the final detection bandwidth. Note that the coil resistance can be written as $R_{c}=\omega _0 L_c/Q_c$, where $L_c$ and $Q_c$ are the coil inductance and quality factor at $\omega _0$, respectively. Also $\Delta f\approx \left( 1/T_{2}^{*}+1/T_{acq}\right)$, where $T_{2}^{*}$ is the decay constant due to inhomogeneous broadening of the NQR line (which determines the width of each spin echo) and $T_{acq}$ is the duration of the echo acquisition windows within each SLSE sequence.

In practice, the SNR obtained after averaging across several scans is reduced by (1) signal decay due to transverse ($T_{2,eff}$) relaxation during each SLSE sequence, and (2) wait times $T_W$ between SLSE sequences to allow for longitudinal ($T_{1}$) relaxation. This effect has been analyzed in detail in our earlier work^18^; the SNR available per echo after averaging is found to be

8 $$\begin{aligned} {SNR_{e,av} = SNR_{e}\frac{\left( 1-e^{-\alpha }\right) ^2\left( 1-e^{-\beta }\right) ^2}{\beta \left( \beta +\alpha \delta \right) }, }\end{aligned}$$

where $\alpha =T_{W}/T_{1}$, $\beta =T_{SLSE}/T_{2,eff}$, and $\delta =T_{1}/T_{2,eff}$ are dimensionless parameters, and $T_{SLSE}=N_{E}T_{E}$ is the total duration of a single SLSE sequence (consisting of $N_{E}$ echo periods, each of length $T_{E}$). Since $T_{2,eff}$ is a function of $\left| B_0\right|$, both $\beta$ and $\delta$ vary across the tag during relaxation-based imaging. Thus, $SNR_{e,av}$ can be written as

9 $$\begin{aligned} {SNR_{e,av} = \frac{SNR_{e}}{l_s}\left[ \left( 1-e^{-\alpha }\right) \int _{-l_s/2}^{l_s/2}{\frac{\left( 1-e^{-\beta (x)}\right) }{\sqrt{\beta (x)\left( \beta (x)+\alpha \delta (x)\right) }}dx}\right] ^2, }\end{aligned}$$

where $l_s$ is the length of the tag. Note that $T_{2}^{*}$ decreases with $\left| B_0\right|$ due to Zeeman broadening of the nuclear energy levels^16^. The detection bandwidth $\Delta _f$ (which is required to estimate both $\sigma _{n}^{2}$ and $SNR_{e}$) should thus be set to its maximum value $\Delta f_{max}\approx \left( 1/T_{2,min}^{*}+1/T_{acq}\right)$ within the sample, where $T_{2,min}^{*}$ is the minimum value of $T_{2,min}^{*}$. For example, our measurements of NaClO$_{3}$ show that $T_{2}^{*}\approx 370$ $\upmu$s at $\left| B_0\right| \approx 0$ and $\approx 68$ $\upmu$s at $\left| B_0\right| =40$ G.

Given the measured relaxation time constants of the tagging material, the term in square brackets in Eq. (9) can be numerically maximized to find the measurement parameters that maximize $SNR_{e,av}$, i.e., to find the optimum values of $T_W$ (which controls $\alpha$) and $T_{SLSE}$ (which controls $\beta$). For NaClO$_3$ at room temperature, we know that (1) $T_{1}\approx 39$ ms, and (2) $T_{2,eff}(\left| B_0\right| )$ increases from approximately 1.4 ms (at $\left| B_0\right| \approx 0$) to 28.0 ms (at $\left| B_0\right| \approx 40$ G) when $T_{E}=600$ $\upmu$s^16^. The optimum parameter values are then found to be $T_{W,opt}=63.5$ ms ($\approx 1.63 T_{1}$) and $T_{SLSE,opt}=16.4$ ms, resulting in $SNR_{e,av}\approx 0.048\times SNR_{e}$.

Based on measured data for 3-bit tag patterns, $SNR_{e,av}\ge 5$ (in voltage units) is sufficient to maximize prediction accuracy (Fig. 4d). Denoting this threshold value as $SNR_{min}$, we finally obtain the required number of scans (i.e., the averaging number) as $N_{av}=\lceil \left( SNR_{min}/SNR_{e,av}\right) ^{2}\rceil$ where $\lceil \cdot \rceil$ denotes the ceiling function and both SNR values are in voltage (i.e., rms) units. Thus, the total time required to read the NQR signature is $T_{meas}=N_{av}T_{scan}$, where $T_{scan}=\left( T_{W}+T_{SLSE}\right)$ is the time per scan.

Our detector coil design has $l_c = 43$ mm, $D_c = 10$ mm, $N_{c}=15$ turns, and $Q_{c}\approx 30$ at the resonant frequency ($\omega _{0}$). Also, our RF receiver has $F\approx 1.41$, which corresponds to a noise figure of $NF=10\log _{10}(F)\approx 1.5$ dB. The analysis described above then predicts that at least $N_{av}=350$ scans are required to obtain $SNR_{min}=5$ from a single cylindrical tag ($l_{s}=12$ mm, diameter $D_{s}=9$ mm, cross-sectional area $A_{s}=\pi D_{s}^2/4$) when the NaClO$_{3}$ weight fraction is $\Phi _{w}=10$%. This result corresponds to a minimum measurement time of $T_{meas}\approx 28$ s, which is in good agreement with the experiments. The weight fraction can be further reduced if needed, but at the cost of an approximately quadratic increase in measurement time.

For the default value of $\Phi _{w}=10$%, the NaClO$_{3}$ content of each tag is 0.036 cm$^{3}$ and 90.5 mg by weight and volume, respectively. These values are close to the practical limit of detection of the proposed NQR-based readout technique for mm-scale tags as long as conventional room-temperature inductive detectors are used. Further improvements in sensitivity are possible by using either a cryogenically cooled detector coil, or alternative detectors such as SQUIDs^27^ or atomic magnetometers^28^.

### Experimental demonstration

We demonstrated the practical application of our material biometrics approach by 3D-printing a complex object that embodies an invisible multi-tag signature. As an example, we used a STL design of “the Statue of Liberty”, as shown in Fig. 5a. A software application was written to automatically embed the signature by modifying a user-supplied STL file. In our case, the signature is localized within a cylinder (diameter $d_1=9$ mm), so the program first removes a small cylindrical region from the center of the object, as shown in Fig. 5b,c. The diameter of the excised region is slightly larger than that of the signature ($d_2\approx 11$ mm), such that the detection coils can be inserted into the annulus during signature read-out. The volumes of the original and modified objects (Fig. 5a,b) were 508.256 cm$^3$ and 491.268 cm$^3$, respectively; thus, the signature region occupies $\approx 3.4\%$ of the object volume. In addition, on average only half of the tags encode an amplitude of ‘1’, so the fractional volume of tagged filament is $\approx 1.7\%$. Also, the size of the signature region is *not* proportional to the size of the object, which means that similar signature regions can be used even for very large objects.

Next, the program generates a second STL file that defines the signature pattern. The two files can then be merged and the entire tagged object printed in one step. Alternatively, the two can be printed separately and finally joined together. For our demonstration, we separately printed signature patterns containing 15 tags; an example is shown in Fig. 5d. Note that while the tagged and untagged filaments generally use the same color for additional security, here we have used different colors to aid visualization (white and green for tagged and untagged filaments, respectively). The final 3D-printed object containing the embedded 15-tag signature is shown in Fig. 5e. The signature region is deeply embedded within the object and thus invisible to optical inspection methods, as mentioned earlier. In addition, there is very little X-ray contrast between tagged and untagged regions, making X-ray read-out of the signature extremely difficult. Finally, the air-filled annulus surrounding the signature region makes it invisible to ultrasound-based inspection as well.

## Discussion

The realization of material biometrics in practical applications and its verification involves several steps. Firstly, a unique signature pattern needs to be created, after which the original design must be modified to insert the pattern. Finally, the modified design should be manufacturable with minimal extra effort. We have already automated most of these steps, thus enabling material biometrics to be directly integrated into industrial-scale additive manufacturing. Signature generation has been streamlined by using a filament extruder to create custom ABS filaments with various concentrations of the tagging compound. We have also written a program to automatically insert signature regions into product designs specified in common digital file formats, as mentioned earlier. Improved versions of the program will have the ability to intelligently decide on the size, tag number, location, and other properties of the signature based on user-specified parameters such as security requirements and manufacturing time/cost.

While this paper has focused on “traditional” fused deposition modeling (FDM) using thermoplastic filaments, our approach can be extended to other additive manufacturing technologies such as material jetting (MJ) and selective laser sintering (SLS). Thus, almost any object which can be 3D printed can now be embedded with an invisible signature that can be read only with a specialized NQR-based scanner. The addition of such complex chemical signatures (which can be object- or batch-specific) makes the tagged objects much more difficult to counterfeit. Moreover, our material biometrics approach is not restricted to plastic objects. Any object that contains one or more additively-manufactured non-conductive parts can be tagged using the proposed technique. The presence of nearby conductive (i.e., metallic) parts does not significantly affect signature read-out unless they completely surround the signature region (thus forming a Faraday shield that blocks RF pulses).

We would like to ensure that use of tagged filament to realize embedded signatures does not significantly alter the functionality of the fabricated object. Fortunately, the relatively low concentrations of tagging compound (10% $\hbox {NaClO}_3$) in the filament ensure that the physical and chemical properties of tagged and untagged plastic are similar to each other. In addition, the proposed embedded signature is an integral part of the object, making it impossible to remove or tamper with without significantly damaging the object itself. Nevertheless, the entropy of the signature should be as high as possible to ensure its unclonability, thus protecting the object from being counterfeited. Such unique and unclonable signatures can be used to track tagged objects throughout their life cycle (manufacturing, distribution, use, and disposal).

Consider a signature containing a total of *NM* tags, arranged in *N* groups of *M* tags each. Such a signature is designed to be read-out in parallel using *N* spatially-sensitive detectors, where each detector can read-out *M* tags using relaxation-based imaging (as shown in Fig. 4a). We assume that each *M*-bit group only uses amplitude-based encoding, as in our experiments. Thus, each group can generate $K^{M}$ unique configurations, where *K* is the number of bits encoded by the signal amplitude of each tag. In addition, each of the *N* detectors can be read-out using a unique echo period $T_{E,i}$, $i\in \{1,2\ldots ,N\}$. Here $T_{E,i}$ is simply the time delay between adjacent RF pulses in an SLSE pulse sequence. SLSE relaxation times $T_{2,eff}$ are strongly dependent on the value of $T_{E,i}$^16^, which can thus serve as an authentication key. Assuming that a total of $N_{TE}$ unique values of $T_{E,i}$ can be used for read-out, the total number of unique configurations for the signature thus becomes

10 $$\begin{aligned} \mathfrak {R}= \left( K^{M}\times N_{TE}\right) ^{N}. \end{aligned}$$

For example, let us assume 1-bit amplitude encoding ($K=1$) with $M=5$ tags within each detector, resulting in a reasonable number of $2^{5}=32$ configurations to be decoded by the proposed ML classifier. The value of $N_{TE}$ depends on the tagging compound; for $\hbox {NaClO}_3$, a reasonable value is $N_{TE}\approx 30$. As a result, a system with $N=5$ detectors can generate $\mathfrak {R}\approx 8.15\times 10^{14}$ unique configurations, corresponding to an entropy of 49.5 bits. An attacker with no knowledge of the authentication key (i.e., the values of $T_{E,i}$) cannot correctly train the ML classifiers required for relaxation-based imaging, and so will be forced to use brute force search to read-out the signature. Since a typical measurement takes $T_{meas}\approx 30$ s ($T_{scan}\approx 80$  ms per scan, $N_{av}\approx 350$) to obtain sufficient SNR, it would take the attacker an average of $(\mathfrak {R}/2)\times T_{meas}=390$  million years to discover the signature. Note that $T_{meas}$ is limited by a physical time constant (the longitudinal relaxation time, $T_1$, of the tagging compound) and so cannot be decreased by the attacker. It is also difficult for attackers to significantly speed-up the signature discovery process by running multiple experimental setups in parallel, since each setup is resource-intensive (requiring a new tagged object, detectors, and spectrometer). Thus, our analysis demonstrates the practical impossibility of reverse-engineering the proposed embedded signatures.

In addition, several methods can be used to further increase the size of the configuration space if desired. These include: (1) additional signal features (such as linewidth, which can be controlled to some degree via manufacturing parameters), (2) tagging compounds with multiple quadrupolar nuclei per molecule (each of which generates independent resonances), and (3) multiple tagging compounds (several $^{35}$Cl compounds are known to exhibit the required field-dependent relaxation properties)^16^. For example, using two tagging compounds, each with two $^{35}$Cl nuclei, increases the effective number of tags by $4\times$. Thus, the signature described above will now embody $\mathfrak {R}\approx 4.4\times 10^{59}$ unique configurations, i.e., an entropy of 198 bits. Finally, while we have focused on additively-manufactured objects in our study, the concept can be extended to other manufacturing processes, as long as NQR-sensitive tags can be embedded into the material.

## Materials and methods

This section first introduces generic properties of the embedded signatures and how they were manufactured for our experiments. Next, the experimental setups are briefly discussed.

### Properties of embedded signatures

The proposed authentication signatures are extracted from NQR spectral features of a set of embedded tags. These features are specific to a particular quadrupolar nucleus, compound, and crystal structure. Their use within customized signatures for NQR-insensitive objects is an example of *extrinsic tagging*^18^. The general idea of extrinsic tagging for signature generation, analysis, and labeling is common in the fields of medicine and chemistry; it is utilized in metabolic incorporation, ingestible medicines, and for labeling proteins. A typical example is the investigation of protein function in living organisms by selective labeling through genetic encoding of fluorescent tags that enable high-resolution imaging^29^. Likewise, RF transponders connected directly to the external surface of a standard-sized capsule can potentially serve as extrinsic tag for validating medication compliance via remote detection of ingested pills inside the digestive tract^30^. Desirable properties of extrinsic tags include (1) being chemically unreactive with the original (untagged) material, and (2) not affecting the primary functions of the tagged object. Additive manufacturing provides a programmable and flexible way of incorporating extrinsic tag-based signatures within complex objects, as demonstrated in this paper.

### Manufacturing of embedded signatures

Figure 1a summarizes the methods used to prepare the embedded signatures. Any convenient part of the object can be used to embed the proposed NQR-sensitive signatures. However, ideally they should be $>1$ mm away from external surfaces to prevent reverse-engineering via visual inspection. By default, the tagged ABS filaments contain 10% (by weight) of $\hbox {NaClO}_3$. They are prepared by mixing 90 grams (g) of ABS pellets with 10 g of $\hbox {NaClO}_3$ crystals. This mixture is then fed into a filament extruder (Felfil Evo). The filament is extruded at $<210\,^\circ$C and spooled on a filament holder to be used in 3D printing.

Experimentally, we observed that the $\hbox {NaClO}_3$ crystals tend to adhere to each other during storage. It is not advisable to directly use these crystals to prepare the mixture, since they tend to clog the nozzle of the filament extruder, which in turn tends to overheat and burn the ABS within the heating chamber. In addition, filament extrusion temperatures $>240\,^\circ$C should be avoided to prevent melting of the $\hbox {NaClO}_3$ crystals. Thus, the $\hbox {NaClO}_3$ is powdered using a grinder (resulting in a typical particle diameter $\approx 40$ $\upmu$m) before being used in filament extrusion. We also observed that the initial extrusion step does not distribute the tag evenly throughout the filament. Thus, the initially-extruded filament is broken into small parts and again fed into the extruder. The result of this second extrusion is a filament with a relatively uniform distribution of $\hbox {NaClO}_3$ that is suitable for printing embeddded signatures.

**Figure 6:**
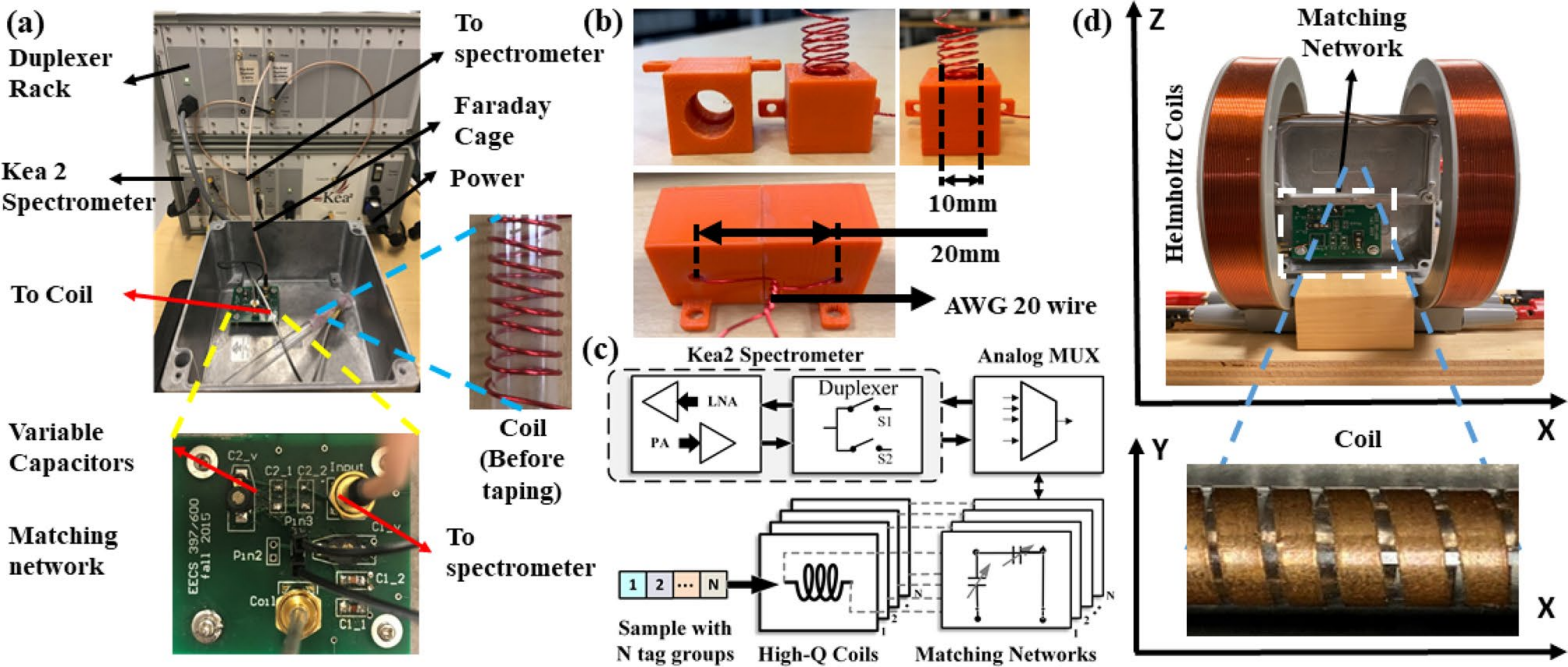
(**a**) The basic experimental setup, which consists of a bench-top MR spectrometer (Magritek Kea2), duplexer rack, impedance matching network, and solenoid coil. (**b**) A single spatially-selective detector (diameter $=10$ mm, length $=20$ mm, 13 turns) wound using AWG 20 copper wire. This particular version was designed to scan small objects (diameter $<10$ mm) that fit within the detector. (**c**) Block diagram of the analog front-end (AFE) used to read-out embedded NQR-active signatures. A total of $N=4$ spatially-selective detectors is shown. Here PA $=$ power amplifier (used to generate RF pulses) and LNA $=$ low-noise amplifier (used to amplify the resulting spin echoes). (**d**) Modified experimental setup used for relaxation-based imaging. A set of Helmholtz coils is used to generate the necessary static field gradient [Microsoft Visio].

### Experimental setups

The basic experimental setup is shown in Fig. 6a. It comprises of the inductive detectors, impedance-matching networks, and a multiplexer mounted inside a Faraday cage to decrease environmental radio frequency interference (RFI). The multiplexer is connected to a commercial bench-top magnetic resonance (MR) spectrometer (Magritek Kea) that contains a power amplifier (PA), low-noise amplifier (LNA), and transmit-receive switch (duplexer). The PA works as the transmitter and the LNA as the receiver, while the duplexer switches the detector between transmit and receive modes. The spectrometer is controlled from a PC via a graphical user interface (GUI) that enables the user to create pulse sequences and store the acquired data.

Typical detectors for NQR spectroscopy use solenoidal coils that are wound on a former (e.g., thin plastic tube) to ensure mechanical stability, as shown in Fig. 6a. Alternatively, coils can be attached to the inner surface of a 3D-printed support, as shown in Fig. 6b. Either geometry can be used for spatially-selective detection. The former allows larger objects to be scanned (by inserting the coil within an annulus, as in Fig. 5). The latter provides higher sensitivity (due to improved fill factor) but is limited to smaller objects that fit within the coil.

A block diagram of the analog front-end (AFE) used to read-out NQR-active signatures is shown in Fig. 6c. The spectrometer, duplexer, multiplexer, and matching networks are similar for all experiments, while the detector coils can be customized based on sample geometry.

The modified experimental setup used for relaxation-based imaging is shown in Fig. 6d. It is similar to the basic setup shown in Fig. 6a, but with the addition of Helmholtz coils to generate the necessary static field gradient across the sample. The currents in each half of the Helmholtz pair are individually set to allow control over both the average $B_0$ field along the *x*-axis (denoted by $\overline{B_{0}}$), and also the field gradient $G_x = \partial B_{x}/\partial x$. The goal is to avoid spatial aliasing by ensuring that each point in the sample has a unique value of $|B_0(x)|=\left| \overline{B_{0}}+G_{x}x\right|$, and thus a unique value of $T_{2,eff}$^16^.

The temperature of the object, $T_s$, can drift during each experiment due to both environmental fluctuations and heating caused by the RF pulses. NQR resonance frequencies are generally temperature-dependent due to changes in both the local electric field gradient (EFG) tensor and vibration modes of the crystal lattice^31^. For ${\hbox {NaClO}_3}$, an approximately linear decrease of $df_{0}/dT_s\approx -5$  kHz/K is observed around room temperature^32^, where $f_0(T_s)=\omega _0(T_s)/(2\pi )$. Controlling the object temperature is difficult since its geometry is not known a priori. Instead, we implemented a tracking loop to compensate for temperature drifts. A program (written in MATLAB) periodically estimates the resonant frequency $f_0(T_s)$ from the echo spectrum, and then commands the spectrometer to adjust its RF excitation frequency to remain on resonance.
